# Zoonotic potential of uropathogenic *Escherichia coli* lineages from companion animals

**DOI:** 10.1186/s13567-025-01493-0

**Published:** 2025-03-26

**Authors:** Nicolas Jousserand, Frédéric Auvray, Camille Chagneau, Laurent Cavalié, Christelle Maurey, Amandine Drut, Rachel Lavoué, Eric Oswald

**Affiliations:** 1 IRSD, Université de Toulouse, INSERM, INRAE, ENVT, UPS, Toulouse, France; 2 Service de Bactériologie-Hygiène, CHU Toulouse, Hôpital Purpan, Toulouse, France; 3 UMR Virologie, INRAE, ENVA, ANSES, Université Paris-Est, Maisons-Alfort, France; 4 Department of Internal Medicine, Ecole Nationale Vétérinaire d’Alfort, Université Paris-Est, Maisons-Alfort, France; 5 Oniris VetAgroBio Nantes, Université de Nantes, Nantes, France; 6 MIHA Team, INRAE, AgroParisTech, Micalis Institute, Université Paris-Saclay, Jouy-en-Josas, France; 7 Present Address: InTheRes, Université de Toulouse, INRAE, ENVT, UPS, Toulouse, France

**Keywords:** Urinary tract infection, uropathogenic *E. coli*, bacteriuria, sequence type, companion animals, dog, cat, zoonosis

## Abstract

*Escherichia coli* is responsible for urinary tract infections (UTI) in humans and pets. This study aims to provide data on the virulome and resistome of *E. coli* strains isolated during bacteriuria in companion animals and to assess their zoonotic potential. 135 *E. coli* strains prospectively collected from urine samples of 44 cats and 91 dogs in three French veterinary teaching hospitals were analyzed via antibiotic susceptibility tests and whole genome sequencing. Phylogroup B2 was overrepresented and several sequence types (STs) associated with human extra-intestinal pathogenic *E. coli* (ExPEC) were found. These included ST12, ST127 and ST141 (8 strains each), which were characterized by genetic homogeneity, and ST73 (23 strains) which contained several serotype-delineated sublineages with distinct distributions in pets and humans. Single nucleotide polymorphism (SNP) analysis further revealed the existence of highly related human and companion animal clones among these STs, indicative of a zoonotic potential. By contrast, other major human ExPEC STs (e.g. ST131, ST10, ST69, ST95 and ST1193) were rarely found (2 strains each), suggesting they might be less adapted to cats and dogs. Of note, ST372 (21 strains) was predominant and exclusively found in dogs. Pet *E. coli* UTI strains carried virulence genes commonly found in human *E. coli* UTI isolates. 15.6% of strains were predicted as multi-drug resistant. The major canine and feline ExPEC lineages were not associated with extended spectrum beta lactamase and AmpC production. Only one strain (from ST131) carried the bla_CTX-M-15_ gene. Persistent clones of *E. coli* isolated from five cats and nine dogs with recurrent infection had genetic traits similar to strains from other animals. Approximately one-third of the *E. coli* UTI strains from pets exhibited genetic similarities to those responsible for UTI in humans, suggesting a potential for zoonotic transmission. This study underscores the continued need to monitor and control antimicrobial resistance in companion animals.

**Supplementary Information:**

The online version contains supplementary material available at 10.1186/s13567-025-01493-0.

## Introduction

Urinary tract infection (UTI) is one of the most common hospitals and community-acquired infectious diseases identified in humans [1], as well as in cats and dogs [2]. Approximately one woman out of two will experience UTI at least once in her life [3], while the incidence in cats and dogs was reported as 19% and 14%, respectively [4, 5]. In the vast majority of cases, the infectious agents involved are bacteria which reside in the gut and ascend the urinary tract, primarily *Escherichia coli*, representing 74% of human UTIs [6] and over 30% in companion animal UTIs [7]. *E. coli* UTI strains have a very wide genetic diversity and carry virulence genes conferring pathogenic properties (adhesion to host cells surfaces, cellular invasion, iron capture, escape from the immune system, etc.). *E. coli* strains responsible for UTI and other extra-intestinal infections in humans and pets often belong to the phylogenetic group B2 [8–11]. Twenty major sequence types (including ST131, ST73 and ST95) account for most human *E. coli* UTI isolates [12]. Major STs have also been identified as a cause of UTI in companion animals, including ST372, ST73, ST127, ST12 and ST141 [8–10, 13]. *E. coli* UTI strains may also carry antimicrobial resistance genes, making them more difficult to treat [11, 14]. This is especially true for highly resistant lineages such as ST131 or ST1193, in contrast to STs exhibiting lower multidrug resistance levels such as ST12, ST73, ST95 and ST127 [12].

In the EU, 44% of households have a companion animal, including over 90% cats or dogs [15]. Given this close physical proximity, pets and their owners can be exposed to pathogenic bacteria originating from each other, especially during an episode of UTI. Several cases of bacterial strain transmissions between humans and pets have been reported [16–20]. The use of antibiotics in companion animals for the treatment of infectious diseases, including urinary tract infections, may select antibiotic resistant strains with zoonotic potential, creating a public health issue [21]. Furthermore, it is possible for companion animals to become infected with *E. coli* strains from their owners. These strains may exhibit resistance to antibiotics that are not used in veterinary medicine and are restricted to human medicine [22, 23]. Such animals may serve as reservoirs for these bacteria, thereby facilitating their spread. Despite the known risk that urinary *E. coli* strains from cats and dogs poses, there is a paucity of data assessing the resistome and virulome of these bacteria in companion animals.

Therefore, we analyzed the genetic diversity of a collection of *E. coli* strains isolated during urinary tract infections in cats and dogs in France, with a particular focus on virulence and antibiotic resistance genes. Additionally, we evaluated the zoonotic potential of these strains based on the genetic information obtained.

## Materials and methods

### Study design and specimen collection

This is an observational, prospective cross-sectional study, conducted between October 2018 and July 2020. Urine collection was obtained by cystocentesis (reference method for urine sampling in veterinary medicine, according to current guidelines), except for 7 cases where this was not possible and a spontaneous micturition was used. Each urine specimen was immediately placed into a plain tube without activator and kept refrigerated at 4 °C. Aerobic quantitative bacterial culture was performed within 12 h after sampling [24]. Bacterial load (CFU/mL) and bacterial species were recorded. The isolated *E. coli* strains were defined here as UPEC strains based on the clinical symptoms observed, i.e. without reference to previous virulence gene-based nomenclatures given the complexity and lack of consensus for UPEC definition.

### Study population

Dogs or cats presented for a consultation or hospitalized at the veterinary teaching hospitals of the National Veterinary Schools of Toulouse, Alfort or Nantes (France), and for which a urinalysis was considered relevant for the medical assessment by the attending clinician, were selected for inclusion. No a priori exclusion criterion was considered. For each dog and cat, the diagnosis of a UTI was established according to international guidelines [25]. UTI included bacterial cystitis (defined as a bacterial infection of the bladder, with compatible lower urinary tract signs, i.e. dysuria, stranguria, pollakiuria, hematuria, along with the presence of bacteria in urine), pyelonephritis (defined as a upper urinary tract infection based on the presence of at least three criteria among the following: pelvic dilation on renal ultrasound examination, azotemia, hyperthermia, neutrophilic leukocytosis and/or increased serum protein inflammation concentration, i.e. C reactive protein in dogs and serum amyloid A in cats) and subclinical bacteriuria (defined as the presence of bacteria in urine in the absence of clinical evidence of infectious urinary tract disease). Animals with a condition that could mimic or impair expression of clinical signs, leading to erroneous identification of clinical expression, were excluded. Urine bacterial culture was performed when lower urinary tract signs or polyuria-polydipsia was present, or when investigation of an infectious process (including pyelonephritis) or a predisposing condition (i.e. kidney disease, endocrine disorder, urinary tract malformation, urolithiasis, current immunosuppressive treatment, nervous disorder) was needed. Animals for which a quantitative culture was negative or considered as non-significant (defined by the growth of < 10^3^ colony-forming unit (CFU)/mL for the samples collected by cystocentesis, and < 10^5^ CFU/mL for the samples collected by other techniques [26]), or positive for a bacterial species other than *E. coli*, were excluded a posteriori.

When a second episode of positive urine culture was identified, the additional *E. coli* strains were compared to the initial isolates from first cultures to describe their potential genetic evolutions.

### Antimicrobial susceptibility profiles

Antimicrobial susceptibility profiles were determined (Vitek 2 system, bioMérieux, France) at the Bacteriology Laboratory of the Toulouse Hospital according to the criteria of the European Committee on Antimicrobial Susceptibility Testing [27]. Sensitivity to penicillins (amoxicillin, amoxicillin-clavulanate, piperacillin-tazobactam), cephalosporins (cefoxitin, cefixime, ceftazidime, ceftriaxone), carbapenem (ertapenem), quinolones (nalidixic acid, ofloxacin), co-trimoxazole, fosfomycin, aminoglycosides (gentamicin, amikacin) and nitrofurantoin were tested.

### Whole genome sequencing and analysis

The Illumina^®^ NextSeq500 instrument (IntegraGen, Evry, France) was used for 2 × 150 bp paired-end sequencing. Following de novo assembly with SPAdes using BioNumerics 7.6 sotware (Biomérieux) and Enterobase [28], genomes were analyzed for identification of sequence type (ST), resistance genes, virulence genes, FimH type and serotype at the Centre for Genomic Epidemiology (CGE) [29], using MLST, ResFinder, VirulenceFinder, FimTyper and SeroTypeFinder, respectively. Strains were assigned to phylogroups using ClermonTyping and EzClermont programs at Enterobase. The presence of virulence and resistance genes was confirmed using ABRicate [30], with Ecoli_VF and Resfinder databases, respectively. A customized database was interrogated to identify virulence-associated genes, alleles or operons lacking in Ecoli_VF database, including *etsABC*, *cdtABC* (types I, II, III, IV and V), *papG* (types I, II and III), *uclD*, *entA*, *sinH*, f17b-A, f17b-G, *clbS*, plasmid-borne *ompT*, *hlyF* and *mig14*, *pdu* operon genes (*adhE*, *astD*, *ccmL*, *ddrA*, *pduA*-*F*, *pduU*, *pduV*, *rhaR* and *rsxC*) and capsule genes *neuA* and *neuC* (K1), *kslB* (K2), *kfiA* and *kfiC* (K5), *catB*-like O-acetyltransferase gene (K14) and *tagB*-like CDP-glycerol glycerophosphotransferase gene (K100) [31].

For phylogenetic analysis of the 135 *E. coli* strains, a Maximum-likelihood algorithm (RAxML)-based tree was obtained using the Enterobase SNP project, annotated and visualized using the iTOL (Interactive Tree of Life) software. For phylogenetic distance analysis, single nucleotide polymorphism (SNP) calling with strict SNP filtering (by removing position with at least one unreliable or ambiguous base or gap, and a minimum absolute 5 × coverage) was performed and minimum spanning trees (MST) were obtained with BioNumerics 7.6, using the following reference strains: *E. coli* K-12 MG1655 (accession: U00096.3) for comparison of 28 *E. coli* strains from pets with recurrent infection; *E. coli* CU532_9 (accession: CP082776.1), *E. coli* CFT073 (accession: AE014075.1), *E. coli* 536 (accession: NC_008253.1), *E. coli* 04–00955 (accession: NZ_CP035498.1) and *E. coli* AO_34/86 (accession: NZ_CP120567.1) for comparison of subsets of 11 ST12, 30 ST73, 15 ST127, 24 ST141 and 5 ST372 human and pet *E. coli* strains, respectively.

Plasmid replicons were identified using ABRicate with PlasmidFinder database. IncF plasmids of *E. coli* strains FT81 and FT115 were assembled from Illumina^®^ reads with BioNumerics 7.6, using plasmid pN17EC0616 nucleotide sequence as a reference [32]. Their FAB type was assigned with pMLST typing scheme at CGE and Blast Ring Image Generator (BRIG) software was used for comparative plasmid analysis.

Metadata, accession numbers, virulence genes, resistance genes and phenotypic resistance profiles are provided for all isolates in Additional file 1.

### Statistical analysis

Chi-square tests were used to compare the proportions of strains or genes between different groups, except when less than 80% of the cells had expected frequencies below 5, in which case Fisher exact tests were employed. *P* < 0.05 were considered significant.

## Results

### Phylogenetic analysis of urinary *E. coli* from dogs and cats

One hundred and thirty-five *E. coli* strains isolated from 91 dogs and 44 cats with bacterial cystitis and/or pyelonephritis or subclinical bacteriuria were included in this study. Additional 14 strains obtained from a second positive specimen from 9 dogs and 5 cats that experienced recurrent infections are described later. Whole genome sequence analysis was performed to investigate the diversity of these 135 *E. coli* strains, including their phylogenetic groups, sequence types, clonal complexes, serotypes and *fimH* types.

**Figure 1:**
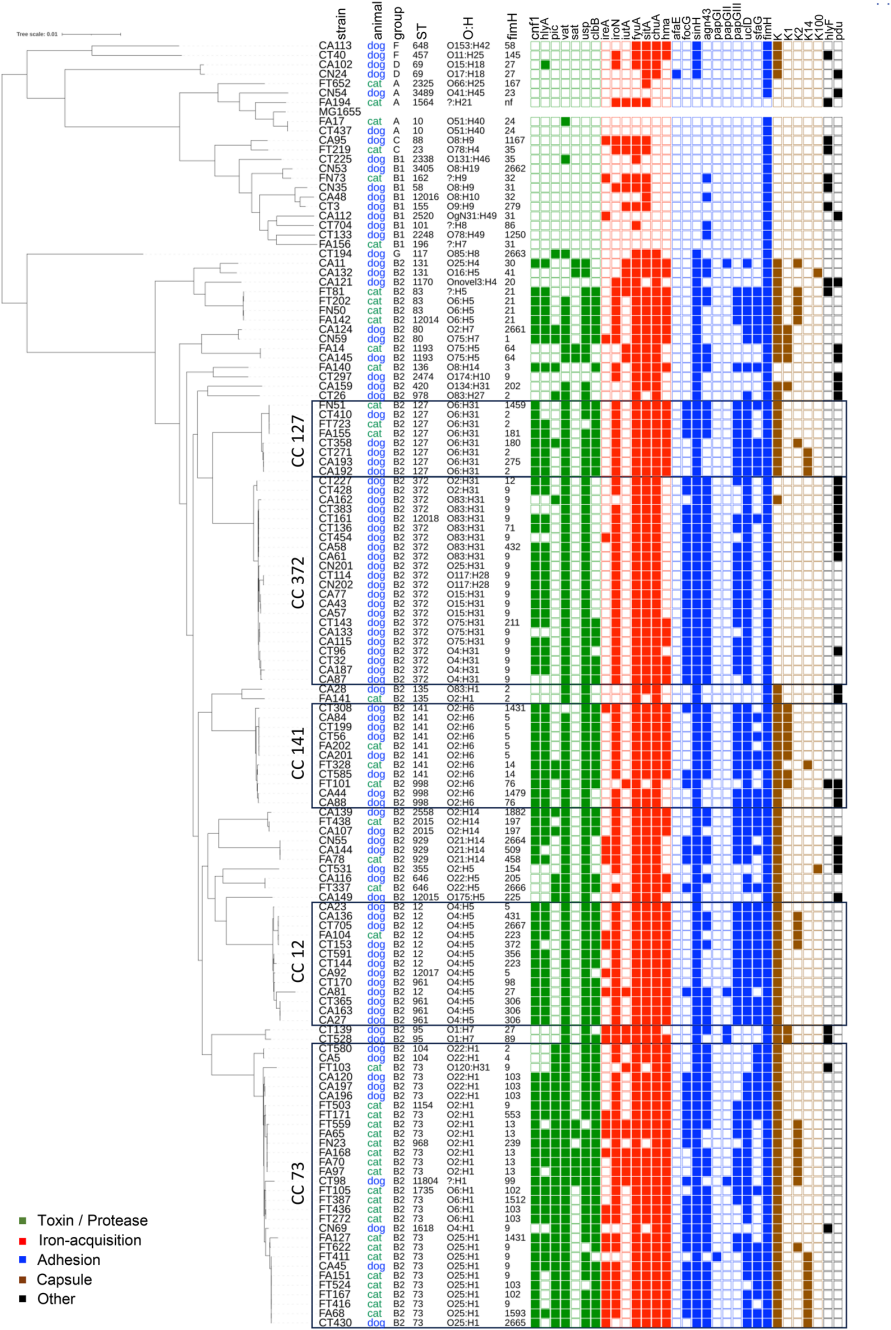
**Phylogenetic distribution and virulence-associated genes of 135 urinary** ***E. coli*** **strains from companion animals.** Strain name, animal origin, phylogenetic group, Sequence Type (ST), serotype (O:H) and *fimH* types are indicated for each isolate. Colored squares represent the presence of a virulence-associated gene. The main clonal complexes are boxed. The *E. coli* strain MG1655 was used as a reference for the phylogenetic analysis.

A large majority of these strains (*n* = 113; 83.7%) belonged to the phylogenetic group B2, in both dogs and cats (82.4% and 86.4%, respectively), followed by B1 (8.8%), F (3.3%), A (2.2%), D (2.2%) and C (1.1%) in dogs, and A (6.8%), B1 (4.5%) and C (2.3%) in cats. The 135 *E. coli* strains were grouped into 53 sequence types (STs) (Figure 1). The most frequent STs belonged to the B2 phylogroup and included ST73 (17%, 23 isolates), ST372 (15.6%, 21 isolates), ST12 (5.9%, 8 isolates), ST141 (5.9%, 8 isolates) and ST127 (5.9%, 8 isolates). Except for ST372, they are considered as major human ExPEC. Other ExPEC STs commonly found in humans, such as ST131, ST69, ST10, ST95 and ST1193, were rarely found here in companion animals since only two isolates were obtained from each of these STs. Taking into account the clonal complexes (defined as groups of STs differing by only one gene from at least one other member of the complex), the most frequent CCs were CC73 (23%, 31 isolates), CC372 (16.3%, 22 isolates), CC12 (9.6%, 13 isolates), CC141 (8.1%, 11 isolates) and CC127 (5.9%, 8 isolates).

When comparing dogs and cats, the most common STs/CCs found in dogs were CC372 (24.2%), CC12 (13.2%), CC73 (11%), CC141 (8.8%) and ST127 (5.5%), while CC73 (47.7%), CC83 (9.1%), CC141 (6.8%) and ST127 (6.8%) were the most frequent STs/CCs in cats. Strikingly, CC372 was exclusively found in dogs, and CC73 was predominantly found in cats, although not exclusively (Figure 1).

The diversity of urinary *E. coli* strains was further analyzed by determining their O:H serotype and type I fimbrial adhesion *fimH* allele. At least 50 distinct serotypes were identified, the most frequent ones being O4:H5 (9.6%), followed by O2:H6 (8.1%) O25:H1 (7.4%), O2:H1 (6.7%), O6:H31 (5.9%) and O83:H31 (5.2%). Some major CCs contained multiple serotypes that formed distinct subclusters in the phylogenetic tree, such as CC372 (which contained O83:H31-, O15:H31-, O75:H31- or O4:H31-linked sublineages) and CC73 (which contained O2:H1-, O6:H1-, O22:H1- and O25:H1-delineated subclusters) (Figure 1). By contrast, the three major CCs CC141, CC12 and CC127 corresponded each to a single serotype, i.e. O2:H6, O4:H5 or O6:H31, respectively. A total of 67 *fimH* alleles were identified, *FimH9* being the most prevalent (20%) allele. Most CC372 isolates contained the *fimH9* allele, whereas the four other major CCs were characterized by a high heterogeneity of *fimH* alleles (Figure 1). No association could be identified between the presence of particular *fimH* alleles and the severity of the disease (Figure 1).

### Virulence-associated genes

The urinary *E. coli* strains were analysed for their repertoire of virulence genes grouped by functional category, including toxins, iron acquisition, adhesion and capsule.

**Figure 2:**
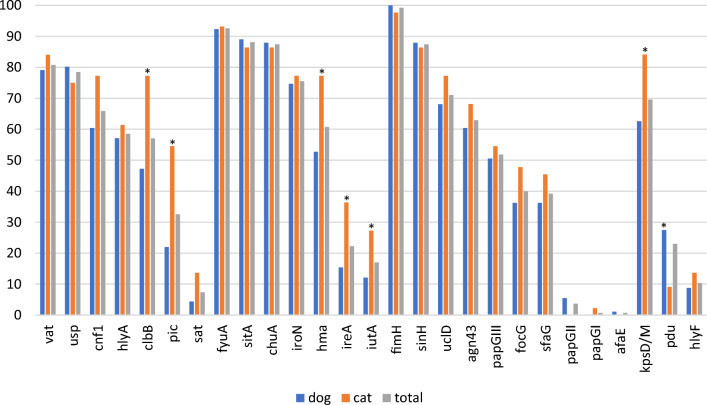
**Distribution of virulence-associated and pdu genes among 135 urinary** ***E. coli*** **isolates from companion animals.** *, gene whose association with dog or cat was considered significant at *p*-value < 0.05.

A high proportion of the urinary *E. coli* strains contained toxin-encoding genes, including *vat* (vacuolating autotransporter toxin; 81%), *usp* (uropathogenic-specific protein; 79%), *cnf1* (cytotoxic necrotizing factor; 66%), *hlyA* (hemolysin; 59%) and *clbB* (colibactin; 57%) (Figure 2). The *pic* (serine protease autotransporter) and *sat* (secreted autotransporter toxin) genes were less frequently detected (33% and 7%, respectively). As clearly observed in Figure 1, the toxin genes were found almost exclusively in B2 strains, the only exceptions being *hlyA*, *pic* and *vat* which were identified in one D (ST69) strain, one G (ST117) strain, and three strains from groups A (ST10), G (ST117) and B1 (ST2338), respectively (Figure 1).

The urinary *E. coli* strains also contained several iron-acquisition genes, especially *fyuA* (yersiniabactin; 93%), *sitA* (iron transport; 88%), *chuA* (heme acquisition; 87%), *iroN* (salmochellin; 76%) and *hma* (heme receptor; 61%) which were the most prevalent (Figure 2). The *ireA* (iron responsive element; 22%) and *iutA* (aerobactin; 17%) genes were less frequent. For the strains of the B2 group, the genes *fyuA*, *chuA*, *sitA* and *iroN* were present in 85% to 100% of the isolates. By contrast, *fyuA* and *sitA* were present in 55% and 59% of the non-B2 isolates, respectively, and the other iron acquisition genes in only 9% to 27% of them (Figure 1).

The most frequently found adhesion genes were *fimH* (type 1 fimbrial adhesin; 99%), *sinH* (invasion-like autotransporter protein; 87%), *uclD* (F17-like Ucl fimbrial adhesin; 71%), *agn43* (autotransporter antigen 43; 63%), *papG*-III (P fimbrial adhesin allele III; 52%), *focG* (F1C fimbriae; 40%) and *sfaG* (S fimbrial adhesin; 39%) (Figure 2). As also seen for toxin-encoding genes, these adhesion genes were mainly found in B2 strains, except for *fimH*, *sinH* and *agn43* which were also present in 96%, 23% and 18% of non-B2 strains, respectively. The remaining adhesion genes *afaE* (afimbrial adhesin), *papG*-I and *papG*-II (P fimbrial adhesin alleles I and II) were rarely detected in the whole collection of strains (1%, 1% and 4%, respectively) (Figure 1).

K capsule genes *KpsD* or *KpsM* were detected in 94 (70%) of the *E. coli* strains, especially in B2 strains (80%) compared to non-B2 strains (18%) (Figure 2). Among the 94 K-positive strains, 47 (50%) could be typed for their K type. K2 was the most common type (18 strains), followed by K1 (15 strains), K14 (12 strains) and K100 (2 strains). The K5 type was not identified.

Other virulence-associated genes with various functions were analysed. These included the *hlyF* gene associated with severe UTI [33] which was detected in 10% of the strains (Figure 2), more frequently in non-B2 (32%) than B2 strains (6%) (Figure 1). The distribution of the propanediol utilization operon *pdu*, which was recently proposed as a contributor to the prominence of certain ST372 sublineages in dogs [34], was found in 29 isolates, mainly from the B2 group (Figure 1). Except for four isolates, the *pdu*-positive strains originated from dogs. They included 10 out of 22 CC372 strains, which corresponded to O2:H31 and O83:H31 strains and one out of the four O4:H31 strains. The other CC372 sublineages, corresponding to O15:H31, O25:H31, O75:H31 and O117:H28 serotypes, lacked the *pdu* genes.

Finally, one B1-ST2338 strain (CT225 from a dog) contained the genes *eae* and *bfpA* (truncated version) and was molecularly defined as EPEC (Additional file 1). Two A-ST10 strains (feline FA17 and canine CT437) contained the *eae* gene only and were defined as atypical EPEC. All three strains carried a complete locus for enterocyte effacement (LEE) with a gamma intimin subtype. Besides, eleven other *E. coli* strains harbored the thermostable toxin-encoding *astA* gene. No Shiga toxin-producing *E. coli* (STEC) strain was identified.

Overall, a combination of six virulence genes (*vat*, *usp*, *sinH*, *chuA*, *fyuA*, and *sitA*) was present in at least 70% of the urinary *E. coli* strains. Half of the isolates (55%) contained the pyelonephritis-associated pilus *papG* gene. Some virulence-associated genes were significantly more frequently present in cats than dogs, such as *clbB*, *pic*, *hma*, *ireA*, *iutA* and *kpsM*/*kpsD* (Figure 2).

### Antimicrobial resistance profiles, antimicrobial resistance genes and mobile genetic elements

**Figure 3:**
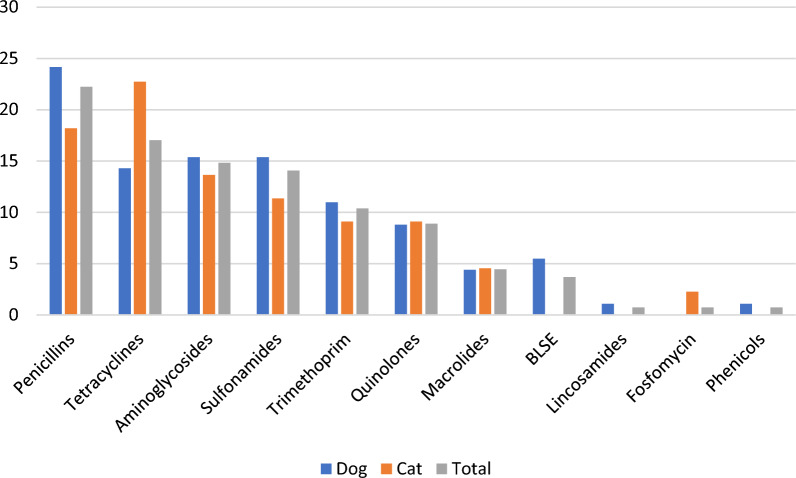
**Distribution of antimicrobial resistance genes among 135 urinary *E. coli* isolates from companion animals.**

**Figure 4:**
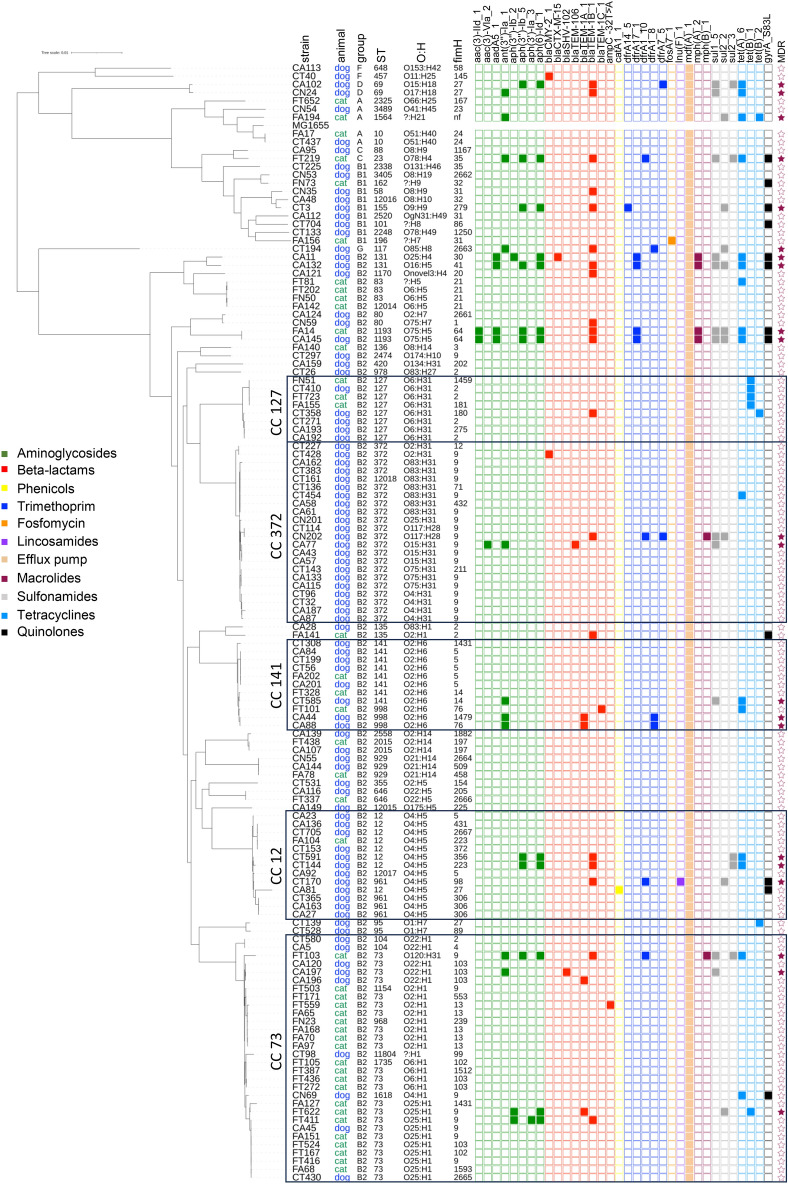
**Phylogenetic distribution and antimicrobial resistance determinants of 135 urinary** ***E. coli *isolates from companion animals.** Colored squares represent the presence of an antimicrobial resistance gene or mutation. Colored stars represent multi-drug resistant (MDR) strains defined as possessing genes or mutations belonging to ≥ 3 distinct antimicrobial resistance categories. Strain name, animal origin, phylogenetic group, serotype (O:H), Sequence type (ST) and *fimH* types are indicated for each isolate. The main clonal complexes are boxed. The *E. coli* strain MG1655 was used as a reference for the phylogenetic analysis.

Resistance of the *E. coli* isolates was tested phenotypically against penicillins, cephalosporins, carbapenems, quinolones, co-trimoxazole (trimethoprim- sulfamethoxazole combination), fosfomycin, aminoglycosides (gentamicin, amikacin) and nitrofurantoin. The highest prevalence of phenotypic resistance was found against penicillins (24%), quinolones (9%) and co-trimoxazoles (8%) (Table 1). The resistance profiles were concordant with those obtained from whole genome analysis (Additional file 1). Genes or mutations conferring resistance to penicillins (22%), sulfonamides (14%), trimethoprim (10%) and quinolones (9%) were among the most frequently detected ones (Figure 3). These included the beta lactamase gene *bla*_TEM-1b_ (14%), sulfonamide resistance genes *sul1* (9%) and *sul2* (7%), trimethoprim resistance genes *dfrA1_10* (3%), *dfrA17_1* (3%), *dfrA1_8* (2%) and *dfrA7_5* (2%) and the quinolone resistance mutation *gyrA*_S83L (9%) (Figure 4).

**Table 1:** **Prevalence of phenotypic antimicrobial resistance among 135 urinary *E. coli* isolates from dogs and cats according to phylogenetic group and sequence type (ST)**

Antibiotic family^a^	Phylogenetic group and/or ST (no. of strains)										
	All (135) (%)	B2 (113) (%)	ST 73 (23) (%)	ST 372 (21) (%)	ST 12 (8) (%)	ST 127 (8) (%)	ST 141 (8) (%)	ST 10 (2) (%)	ST 80 (2) (%)	ST 131 (2) (%)	ST 1193 (2) (%)
Penicillins	24	23	26	24	25	13	0	0	50	100	100
Cephalosporins	3	3	4	5	0	0	0	0	0	50	0
C1G & C2G	2	2	4	5	0	0	0	0	0	0	0
C3G & C4G	3	3	4	5	0	0	0	0	0	50	0
Carbapenems	0	0	0	0	0	0	0	0	0	0	0
Quinolones	9	7	0	0	13	0	0	0	0	100	100
AMG	2	3	0	5	0	0	0	0	0	0	100
SXT	8	6	4	5	0	0	0	0	0	100	100
Fosfomycin	0	0	0	0	0	0	0	0	0	0	0
Nitrofurantoin	1	1	0	0	0	0	0	0	0	0	0

^a^C1G, C2G, C3G and C4G, cephalosporin of first, second, third and fourth generation, respectively; AMG, aminoglycosides (gentamicin, amikacin); SXT, co-trimoxazole (trimethoprim-sulfamethoxazole combination).

Resistance against tetracyclines and aminoglycosides was also predicted through genome analysis for 17% and 15% of isolates, respectively (Figure 3). Tetracycline resistance genes included *tetA* (12%) and *tetB* (4%) (Figure 4). For the aminoglycoside family, the three genes *aph(6)-Id*, *aph(3'')-Ib* and *ant(3'')-Ia* conferring resistance to streptomycin were more frequently detected (i.e. 9%, 7% and 7%, respectively) than the *aac(3)* variant genes conferring resistance to gentamicin (i.e. < 2%) (Figure 4).

**Figure 5:**
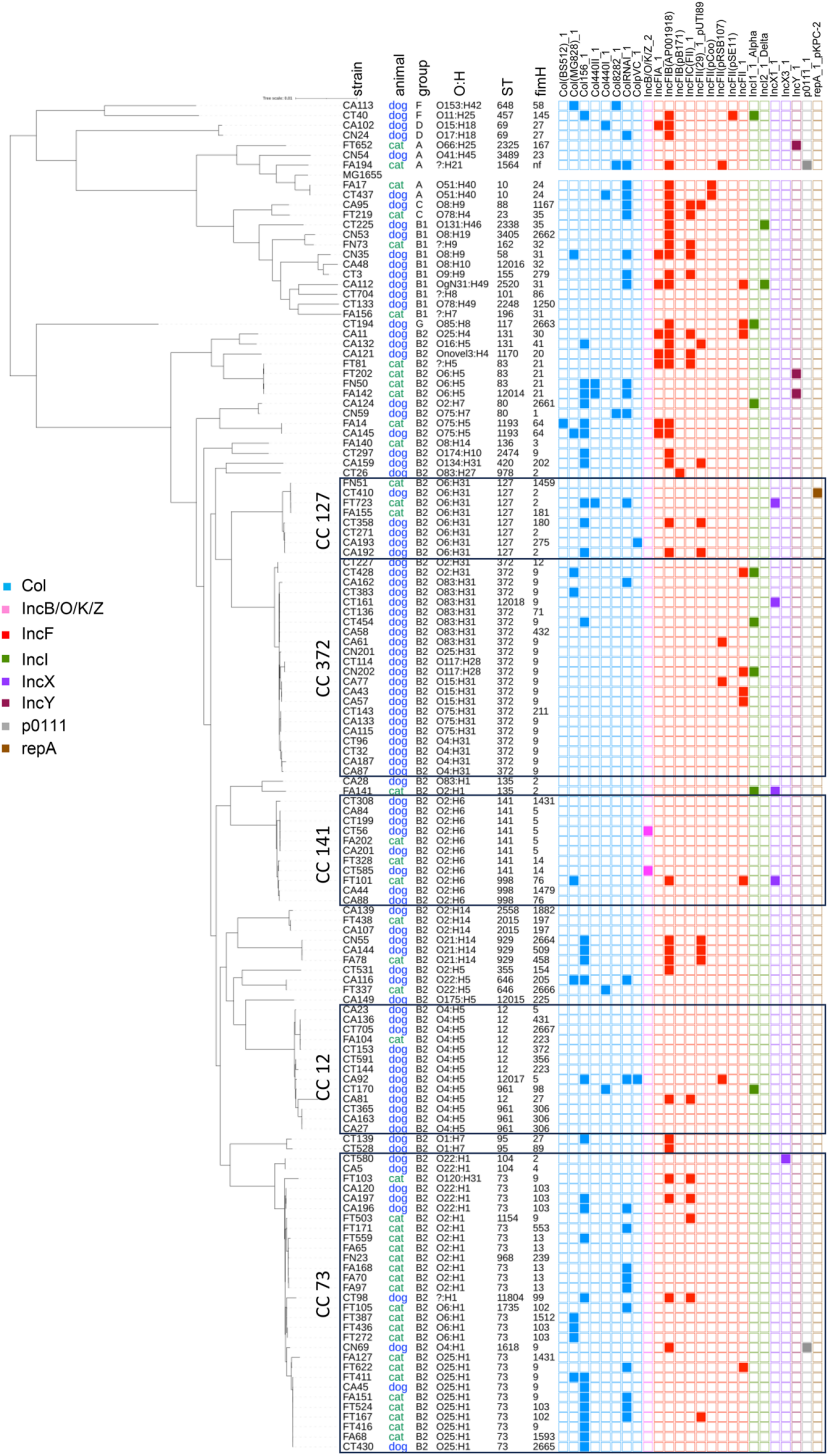
**Phylogenetic distribution and plasmid types of 135 urinary** ***E. coli*** **isolates from companion animals.** Colored squares represent the presence of a plasmid replication origin examined. Strain name, animal origin, phylogenetic group, serotype (O:H), Sequence type (ST) and *fimH* types are indicated for each isolate. The main clonal complexes are boxed. The *E. coli* strain MG1655 was used as a reference for the phylogenetic analysis.

Regarding extended spectrum beta lactamases (ESBL), the *bla*_CTX-M-15_, *bla*_TEM-106_ and *bla*_SHV-102_ genes were identified in only three strains: one B2-ST131 fimH30 isolate (CA11 strain, carrying IncFIA, FIB, FIC and FII replicons), one B2-ST372 isolate (CA77 strain, carrying Inc type FII replicon) and one B2-ST73 isolate (CA197 strain, carrying IncFIB and IncFIC replicons), respectively (Figures 4 and 5). The AmpC-type beta lactamase *bla*_CMY-2_ gene was identified in two isolates belonging to B2-ST372 (CT428 strain, carrying IncFII and IncI replicons) and F-ST457 (CT40 strain, carrying IncFIB and IncFII replicons) (Figures 4 and 5). Finally, a mutation in the *ampC* promoter conferring resistance to cefoxitin, cefotaxime, ampicillin, ampicillin / clavulanic acid and ceftazidime was identified in one B2-ST73 strain (FT559) (Figure 4).

Within each of the five STs most frequently found in companion animals (i.e. ST12, ST73, ST127, ST141 and ST372), the presence of resistance genes or mutations was limited to only a subset of strains (Figure 4), suggesting a recent acquisition. Since plasmids often carry resistance genes, their distribution was also analyzed within each of these major STs and showed heterogeneity (Figure 5). The most frequent replicon types were Col156 (20%), followed by ColRNA (15%) and IncFIB (9.4%). Among the remaining strains of the collection belonging to less frequent STs, resistance genes or mutations were concentrated in a few isolates, especially those belonging to B1-ST155, C-ST23, D-ST69, G-ST117, B2-ST131 and B2-ST1193 (Figure 4), and the main plasmid replicons corresponded to IncFIB (58%), Col156 (28%), ColRNA (26%), IncFIA (16%) and IncFIC (16%) (Figure 5).

A total of 21 (15.6%) *E. coli* isolates could be predicted as MDR based on the presence of genes and mutations conferring resistance to at least three antibiotic families (Figure 4). Carbapenems are not authorized in the EU for animal use and all strains were susceptible to carbapenems. No significant difference was found in antimicrobial resistance prevalence between cats and dogs. Strains from animals having received an antibiotic treatment during the past 6 months were more frequently resistant to antibiotics, although this was not statistically significant (Additional file 3).

### Clinical presentation

**Figure 6:**
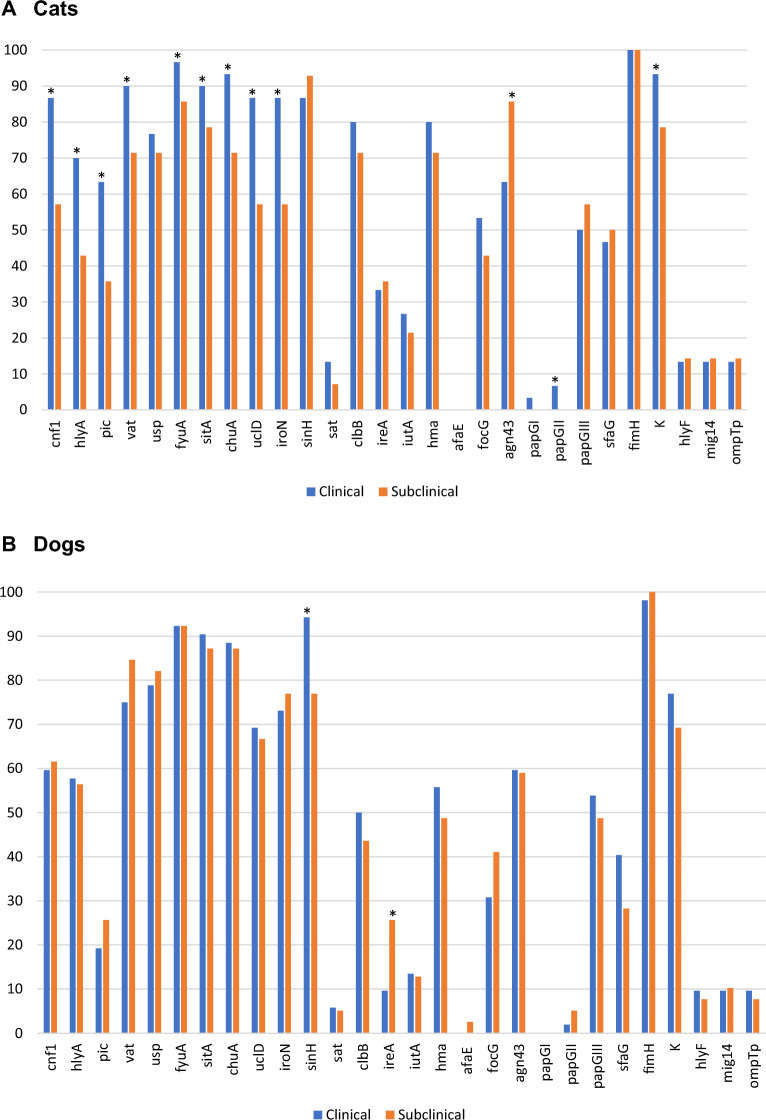
**Distribution of virulence genes among 44 feline (A) and and 91 canine (B)** ***E. coli*** **strains according to symptoms.** These urinary strains originated from individuals with either clinical (i.e. bacterial cystitis, pyelonephritis) or subclinical bacteriuria. *, the difference was considered significant at *p* < 0.05.

Virulence-associated genes were compared between pets with clinical bacteriuria (bacterial cystitis and/or pyelonephritis) and those with subclinical bacteriuria. For cats, 11 genes encoding toxins (*cnf1*, *hlyA*, *pic* and *vat*), iron-acquisition systems (*fyuA*, *sitA*, *chuA*, *uclD* and *iroN*), adhesin (*papG*-II) and K capsule were significantly found more frequently in individuals with clinical manifestations, while it was the opposite for the adhesion gene *agn43* (Figure 6A). For dogs, the invasin-like autotransporter gene *sinH* and the iron responsive element gene *ireA* were significantly more or less frequent, respectively, in individuals with clinical manifestations than in those with subclinical bacteriuria (Figure 6B).

### Analysis of *E. coli* isolates responsible for recurrent infections

**Figure 7:**
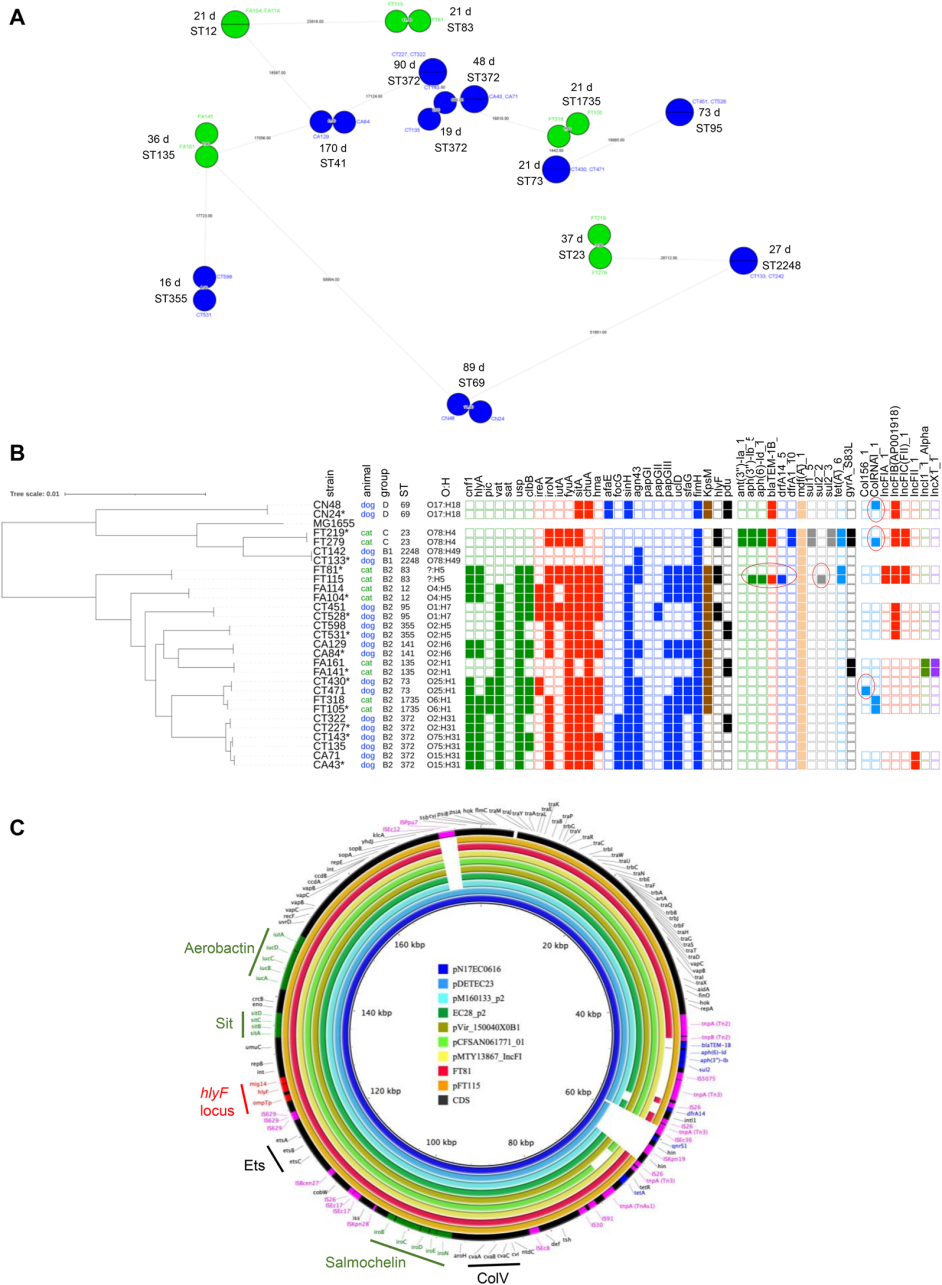
**Genetic comparison of 14** ***E. coli*** **isolate pairs collected each from the same companion animal. A** Minimum spanning tree based on Single Nucleotide Polymorphism (SNP) analysis. Blue and green colors correspond to dog and cat isolates, respectively. Each node represents a unique SNP profile. The node size reflects the number of isolates. The numbers of SNP differences are indicated on the connecting lines between the nodes. For each pair of related strains, the number of days (d) between their isolation and the sequence types (ST) are indicated. **B** Phylogenetic distribution and identification of genetic traits (virulence-associated genes, left; antimicrobial resistance genes or mutations, middle; plasmid types, right). Squares are indicated with color codes as displayed in Figures 1, 4 and 5. Ellipses show the differences between isolates belonging to 4 pairs of strains. Strain MG1655 was used as a reference. Isolates included in the collection of 135 *E. coli* strains characterized in this study are indicated with an asterisk. **C** Blast Ring Image Generator (BRIG) comparison of ColV plasmids from FT81 and FT115 *E. coli* strains with seven related ColV plasmids identified from Genbank with high percentages of coverage and identity. The 179 kb-long plasmid pN17EC0616 (accession: CP043737) from a fluoroquinolone-resistant *E. coli* strain isolated from a chicken leg in the USA [32] was used as a reference. Plasmids are arranged from the inside to the outside as follow: pDETEC23 (accession: CP116101; source: *E. coli* ST1236 strain DETEC-P881, ICU patient, China), pM160133_p2 (accession: CP022166; source: urinary *E. coli* ST1485 strain, New York), EC28_p2 (accession: NZ_CP049102; source: urinary *E. coli* ST429 strain, China), pVir_150040X0B1 (accession: CP104032; source: non-phage-resistant *Klebsiella pneumoniae* strain 150040X0B1, China), pCFSAN061771_01 (accession: CP042897; source: *E. coli* ST1485 strain isolated from a raw milk cheese, Egypt), pMTY13867_IncFI (accession: AP025658; source: carbapenem-resistant *E. coli* strain TUM13867, Japan), pFT81 and pFT115. The outer black circle corresponds to pNE17EC0616 plasmid annotation, with mobile genetic elements (transposon genes, insertion sequences [IS]) highlighted in magenta, resistance genes in blue, iron acquisition and transport systems in green, and the *hlyF* locus in red. On both the outer and inner circumference of the ring, long and short tick marks indicate 500 and 100 kilobase pair increments, respectively.

Recurrent UTIs are common in cats and dogs and can be caused by a persistence of the same bacterial strain or by a reinfection with a different strain or microorganism. In this study, a second positive *E. coli* culture was obtained during the follow-up of 14 pets (i.e. nine dogs and five cats), with an interval ranging from 16 to 170 days between the two samples collected (median of 31.5 days) (Figure 7A). Among these recurrent urinary *E. coli* strains, the phylogroup most frequently found was B2 (79%), as for the whole collection. Various STs were found, including ST73, ST372, ST12, ST141 and ST83. No particular genetic profile that could explain the persistence of these strains overtime was identified.

For each of the 14 pets, the second isolate could be considered as the same as the first one based on SNP analysis (from 0 to 11 SNP identified) (Figure 7A). As expected, both isolates of each pair shared the same phylogroup, ST, virulence genes, antimicrobial resistance genes and plasmid replicons, except for four pairs of strains. Three pairs of isolates showed differences for Col156 or ColRNA plasmids that were gained for each of them (Figure 7B). Interestingly, the fourth pair of *E. coli* strains, FT81 and FT115 (B2-ST83), isolated 21 days apart from a cat with subclinical bacteriuria (that had not received any treatment), showed a difference in five resistance genes, namely *aph(3'')-Ib* (*strA*) and *aph(6)-Id* (*strB*) encoding resistance to streptomycin, *bla*_TEM-1b_ encoding resistance to penicillin, *sul2* encoding resistance to sulfonamide, and *dfrA14* encoding resistance to trimethoprim, which were all lacking in the first strain isolated (FT81) but present in the second one (FT115) (Figure 7B). A large IncF plasmid assigned to the F18:A-:B1 plasmid replicon type was found which was identical in both isolates, except for the five aforementioned resistance genes that were present in the 171,441 bp-long plasmid pFT115 but absent in the 160,666 bp-long pFT81 plasmid (Figure 7C), suggesting a gain of these five genes over time. On pFT115 and similar plasmids retrieved from Genbank, these five resistance genes clustered together within a multidrug-resistance region containing several mobile genetic elements. This region included the transposon *tnpA*/*tnpR* genes and IS5075 element framing the cassette [*bla*_TEM-1b_, *aph(6)-Id* (*strB*), *aph(3'')-Ib* (*strA*) and *sul2*] on one side, and a class I integron (*intI1*) with a unique *dfrA14* gene framed by two IS26 (Figure 7C). By contrast, the pFT81 plasmid lacked these five resistance genes as well as most of the mobile genetic elements (Figure 7C). Besides resistance genes, this large IncF plasmid possessed typical traits of ColV plasmids, including carriage of a colicin V-encoding operon (*cvaABC*, *cvi*), a conjugation system (*tra* genes), iron acquisition and transport operons (*sitABCD*, aerobactin *iutA*/*iucABCD* and salmochelin *iroBCDEN*), an ABC transport system (*etsABC*) and the *tsh* (temperature-sensitive hemagglutinin) and *iss* (serum resistance) genes. It also harbored a locus containing the *hlyF* gene, the putative antimicrobial resistance factor *mig*-14 ortholog gene and the protease ompTp gene, as previously described for other *hlyF*-positive UPEC plasmids [33]. All these genes were conserved in the various ColV plasmids investigated here, in contrast to the resistance genes which showed variability (Figure 7C).

### Phylogenetic distance between companion animal and human UPEC strains

**Figure 8:**
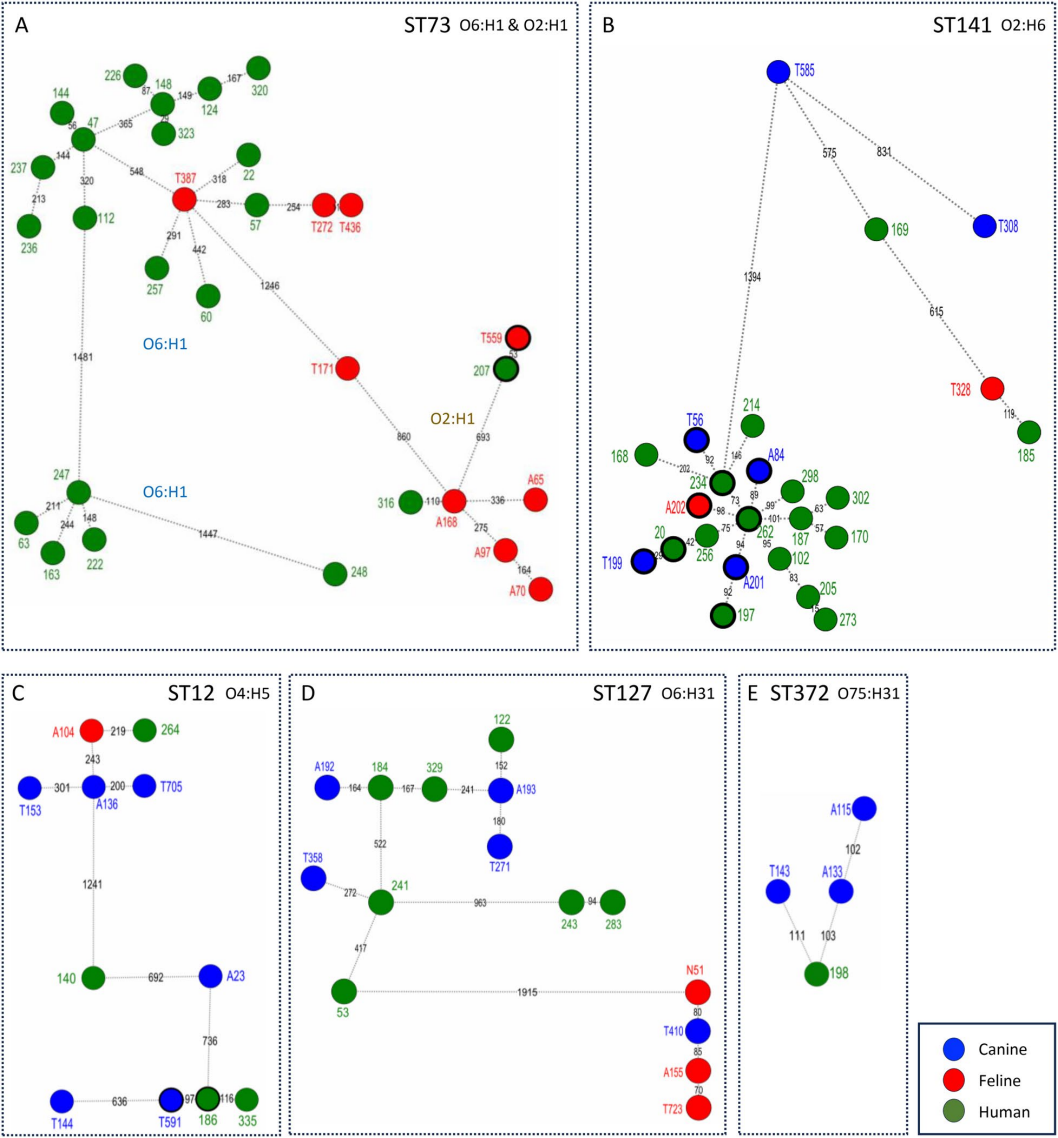
**Single Nucleotide Polymorphism (SNP) distances between human and companion animal UPEC strains.** Minimum spanning trees based on SNP analysis of strains belonging to five major companion animal STs (**A** ST73; **B** ST141; **C** ST12; **D** ST127; **E** ST372) common with humans. Each node represents a strain with a unique SNP profile. The numbers on the connecting dotted lines represent genetic distances (number of differing SNPs) between adjacent nodes. Nodes are coloured according to the source of isolation as indicated in the legend. Nodes with thick (black) outer circles represent strains belonging to mixed pairs of human and companion animal isolates with a distance < 100 SNPs. The prefixes “ECC”, “C” and “F” have been omitted from the names of human, canine and feline isolates, respectively.

Since major human ExPEC ST12, ST73, ST127 and ST141 were identified in dogs and cats and could thus represent zoonotic (or anthropozoonotic) lineages, we analysed the phylogenetic distance between canine / feline strains and isolates from a collection of human UPEC strains isolated in France [33]. A single nucleotide polymorphism (SNP) analysis was performed that was restricted to STs and serotypes common between the two collections of strains, including ST12 (O4:H5), ST73 (O2:H1, O6:H1), ST127 (O6:H31), ST141 (O2:H6) and ST372 (O75:H31). This revealed the existence of highly related clones between companion animals and humans, which exhibited SNP distances < 100 SNPs (Figure 8), a threshold recently used for seeking evidence of potential cross-source linkage [35]. This was especially true for ST141 where six pet/human isolate pairs with a SNP distance ranging from 29 to 98 SNPs were found, and to a lower extent, for ST12 (one pair; distance of 97 SNPs) and ST73 (one pair; distance of 53 SNPs). For ST127 and ST372, closely related genomes could also be identified, but with a distance slightly above the 100 SNPs threshold (e.g. 103 and 111 SNPs [Figure 8E], 152 and 164 SNPs [Figure 8D]).

## Discussion

This study highlights that UPEC strains in cats and dogs gather various lineages and sublineages, some of which are restricted or adapted to cats and dogs, while others are shared with humans and might thus be zoonotic.

As described for humans [36], the phylogenetic group B2 was predominant (83.7%) among the *E. coli* strains that caused bacteriuria in cats and dogs. Indeed, UPEC strains, and notably those belonging to the B2 phylogroup, possess a broad range of virulence factors (as discussed below) allowing them to successfully colonize the urinary tract. Five major STs were identified in pets, i.e. ST372, ST73, ST12, ST127 and ST141. The last four STs have been reported as commonly isolated in humans [12, 37, 38] and could thus be zoonotic or subject to bidirectional transmission. Notably, ST141 (O2:H6) and to a lower extent ST73 (O2:H1) and ST12 (O4:H5), exhibited cross human-pet linkage with SNPs distance < 100 SNPs. This close linkage between human and companion animal isolates likely reflects the global dissemination of UPEC lineages with a zoonotic (or anthropozoonotic) potential, as described recently for ST127 [39]. Strikingly, some STs known as prominent among human ExPEC, such as ST131, ST69, ST10, ST95 and ST1193, were rarely found in pets, as also observed elsewhere [9, 10, 13, 40–42]. For these particular STs, companion animals could thus be considered as spillover hosts rather than primary reservoirs [43].

Remarkably, ST372 was a major ST exclusively found in dogs, as reported previously [8, 10, 13, 36, 42]. ST372 contained multiple serotype-delineated subclusters, including O2:H31, O4:H31, O15:H31, O75:H31, O83:H31 and O117:H28, as previously found [34, 36]. Unsurprisingly, none of the ST372 O18:H31 and O45:H31 sublineages previously described as restricted to human sources [34] were found here, further supporting the existence of distinct human and canine-adapted ST372 sublineages. Among the factors hypothesized to contribute to the evolution and selection of particular ST372 sublineages in dogs was the *pdu* operon involved in the metabolism of glycerol and 1,2 propanediol (i.e. propylene glycol) [34]. These additives are commonly used in semi-moist and soft-moist dog foods and treats, where they act as humectants and help extend shelf life and stability without spoiling or hardening. Concordantly, this operon was also found here in ST372, although its distribution was limited to certain specific ST372 sublineages corresponding to serotypes O2:H31 and O83:H31. The *pdu* genes were also found in non-ST372 strains, such as canine or feline ST135, ST929, ST998 and ST1193 isolates, suggesting a wider dissemination of this operon across the *E. coli* species than originally thought. The absence of *pdu* genes in sublineages accounting for half of ST372 dog isolates also suggests that other genetic traits must be involved in dog adaptation of ST372 strains.

In cats, ST73 was by far the most predominant ST, as described previously [9, 41]. Although ST73 has usually been considered as human-associated, its high prevalence in cats, and also in dogs, suggests that it has a rather ubiquitous nature, as previously reported [10]. This might be explained by the existence of various sublineages within ST73 that could have distinct host adaptation abilities. Indeed, the distribution of ST73 sublineages varied largely between humans and companion animals, as illustrated by the predominance of O6:H1 (70%) and absence of O25:H1 among human ST73 strains, while these serotypes were scarce (13%) or predominant (44%), respectively, among companion animal ST73 strains (Additional file 4).

Virulence genes identified in companion animal UPEC strains included toxin-encoding genes (*vat*, *usp*, *cnf1*, *hlyA* and *clbB*), iron-acquisition systems (*fyuA*, *sitA*, *chuA*, *iroN* and *hma* genes), adhesion genes (*fimH*, *sinH*, *uclD*, *agn43*, and *papG*-III) and K capsule genes. These genes were also commonly found in a collection of 225 human UPEC strains recently described [33]. Some variations were observed however between animal and human *E. coli* strains. The genes *vat*, *usp*, *cnf1*, *hlyA*, *clbB*, *pic*, *iroN*, *uclD*, *agn43*, *papG*-III, *focG*, *sfaG* and *pdu* were significantly more prevalent in companion animal than human *E. coli* strains, while it was the opposite for *sat*, *iutA*, *papG*-II, *afaE*, *kpsD/M* and *hlyF* (Additional file 5). A higher prevalence of *papG*-III in pet than human *E. coli* strains was previously described [41, 44]. These variations in the combinations of virulence genes are likely related to the circulation of distinct UPEC (sub) lineages in humans and pets. Indeed, ST95, ST69, ST131 and ST404 (carrying *sat*, *iutA* and/or *papG*-II genes more frequently than other STs) altogether accounted for 40% of human strains but less than 5% of companion animal isolates (Additional file 6). Furthermore, a more frequent carriage of *sat*, *hlyA*, *iutA* and/or *papG*-II genes was observed in human than companion animal strains (Additional file 2) which might be also attributed to distinct ST73 sublineages colonizing humans and pets, as mentioned above.

This study also showed that *E. coli* strains responsible for UTIs in cats and dogs have a high propensity to cause recurrent infections. Persistence of UPEC clones was observed for a long time within pet hosts, i.e. up to 170 days, as recently described for dogs [45]. As for the whole collection, the recurrent UPEC clones mainly belonged to the phylogroup B2 (79%) and corresponded to various STs, including the major ST73, ST372, ST12 and ST141. It is tempting to speculate that these clones efficiently colonized the intestinal tract of those animals, similar to the proficient gut colonizer *E. coli* ST131 [46]. The prevalent type 1 fimbriae might contribute to efficient intestinal colonization, as reported for ST131. Therapeutics aimed at lowering gut colonization of major ExPEC clones in cats and dogs should thus be considered as interesting strategies in order to reduce UTI in pets. Alternatively, recurrent UTIs might be caused by UPEC that establish quiescent intracellular reservoirs in the bladder and serve as seeds to initiate a new infection [47]. Interestingly, one recurrent feline *E. coli* ST83 isolate showed acquisition overtime of five antibiotic resistance genes on a *hlyF*-positive ColV-like plasmid that is widely spread among UPEC [33], illustrating the central role of plasmids in resistance gene capture and dissemination [48, 49].

Finally, this study showed that resistance to penicillins, tetracyclines, sulfonamides, aminoglycosides and quinolones were the most frequent, as reported previously [10, 42] and 15.6% of the isolates were predicted as MDR. A low prevalence (4.4%) of extended-spectrum cephalosporin (ESC) resistance genes was found among canine and feline UPEC strains, as also described recently for dogs and/or cats [10, 42]. These results reflect the scarcity in pets of ExPEC lineages known to be associated with MDR and ESBLs, such as ST131 and ST1193 [12]. The *bla*_CTX-M-15_ gene was identified only once here, within a dog isolate corresponding to the human-associated ST131/H30 clone which also possesses fluoroquinolone resistance. This result is consistent with the low prevalence of ST131/H30 clone previously reported for dogs in France [10] and with the fact that *bla*_CTX-M-15_ was reported as the most frequent type among ESC-resistant ST131 pet isolates in France [50]. The second ST131 strain isolated here was a ST131/H41 clone that did not carry any CTX-M type gene. Only four other ESC-resistant strains were identified, including one ST372 isolate carrying *bla*_TEM-106_, one ST73 isolate carrying *bla*_SHV-102_ and two (ST372 and ST457) isolates carrying the AmpC *bla*_CMY-2_ gene. This latter gene was also previously reported in pets in France, with a prevalence of 23.4% among ESC-resistant ExPEC [50]. A mutated *ampC* promoter conferring resistance to extended spectrum cephalosporins was only observed once, in a ST998 (CC141) isolate obtained from a cat. This low prevalence of ESC resistant strains and absence of carbapenem-resistant strain might result from the implementation in France, since 2011, of successive national plans (named “EcoAntibio”) aimed at reducing the risks of antibiotic resistance in veterinary medicine. Consequently, the use of third- and fourth-generation cephalosporins in cats and dogs decreased by 65.8% between 2013 and 2022 [51]. Several molecules such as carbapenem are also now prohibited for animal use. Dogs and cats with subclinical bacteriuria are no longer treated. Moreover, the recommended duration of treatment was reduced to limit emergence of antimicrobial resistant strain. For example, 7–14 days antimicrobial cure is now preferred rather than 4–6 weeks treatment previously recommended for acute pyelonephritis.

In conclusion, most urinary *E. coli* strains collected from cats and dogs in France belonged to the phylogenetic group B2, which included the five major lineages ST73, ST372, ST12, ST141 and ST127. The fact that some of these STs are shared between humans and pets suggests the existence of a dynamic circulation between each other, while other STs appear to be restricted to companion animals such as ST372 in dogs. Although the major canine and feline ExPEC clones identified here were not associated with ESBL and AmpC production, and human high-risk ExPEC lineages such as ST131 and ST1193 were scarce, the existence of pet *E. coli* strains with a zoonotic potential indicates that efforts to monitor and control antimicrobial resistance in pets should be maintained.

## Supplementary Information

**Additional file 1. Metadata, accession numbers, virulence genes (Pets_virulence), resistance genes (Pets_resistance) and plasmid types (Pets_plasmids) of 135 urinary strains from cats and dogs, with additional characteristics of 14 recurrent pet urinary strains (Pets_Recurrent) and 225 human UPEC strains (Humans_vir_res_plasmids).**

**Additional file 2. Prevalence of virulence-associated genes in ST73 UPEC strains isolated from humans and companion animals.**

**Additional file 3. Distribution (%) of antimicrobial resistance phenotypes among urinary** ***E. coli*** **strains isolated from animals having received (ATB+) or not (ATB-) an antibiotic treatment during the past six months**. Pen, penicillins; Ceph, cephalosporins; C1G/C2G, cephalosporins of first and second generations; C3G/C4G, cephalosporins of third and fourth generations; QL, quinolones; AMG, aminoglycosides, SXT, co-trimoxazole; Fos, fosfomycin; NFT, nitrofurantoin.

**Additional file 4. Distribution (%) of major serotypes among urinary** ***E. coli*** **ST73 strains isolated from humans (A) and companion animals (B), respectively**.

**Additional file 5. Distribution (%) of virulence-associated genes among urinary** ***E. coli*** **strains isolated from 225 humans, 44 cats and 91 dogs.** *, the difference between human and companion animal *E. coli* strains was considered significant at *p*-value <0.05.

**Additional file 6. Distribution (%) of sequence types among 225 and 135 urinary** ***E. coli*** **isolates from humans (A) and companion animals (B), respectively.**

## Data Availability

Sequencing data for the dog and cat *E. coli* strains are available in the NCBI Database BioProject [52], under the Bioproject number PRJNA1125335.
