# Supplementary material for: Zoonotic potential of uropathogenic Escherichia coli lineages from companion animals
Source: Vet Res. 2025 Mar 26;56:69. doi: 10.1186/s13567-025-01493-0 (PMC11948896; doi:10.1186/s13567-025-01493-0)
Supplement: Supplementary file 2 — Additional file 2. Prevalence of virulence-associated genes in ST73 UPEC strains isolated from humans and companion animals. [file 13567_2025_1493_MOESM2_ESM.docx]

**Additional file 2** **Prevalence of virulence-associated genes in ST73 UPEC strains isolated from humans and companion animals.**

| **Virulence gene** | **Origin of B2 ST73** | | ***p*-value** |
| --- | --- | --- | --- |
|  | **Human**  **N (%)** | **Companion N (%)** |  |
| **Toxins:** | | | |
| *vat* | 26 (96) | 23 (100) | 1 |
| *usp* | 26 (96) | 20 (87) | 0.322 |
| *cnf1* | 25 (93) | 22 (96) | 1 |
| *hlyA ** | 26 (96) | 15 (65) | 0.002 |
| *clbB* | 27 (100) | 22 (96) | 0.46 |
| *pic* | 26 (96) | 23 (100) | 1 |
| *sat ** | 22 (82) | 5 (22) | <0.001 |
| **Siderophores:** | | | |
| *fyuA* | 27 (100) | 23 (100) | - |
| *sitA* | 27 (100) | 22 (96) | 0.46 |
| *chuA* | 27 (100) | 23 (100) | - |
| *iroN* | 23 (85) | 20 (87) | 1 |
| *hma* | 26 (96) | 23 (100) | 1 |
| *ireA* | 14 (52) | 15 (65) | 0.34 |
| *iutA ** | 22 (82) | 6 (26) | <0.001 |
| **Adhesins:** | | | |
| *fimH* | 27 (100) | 23 (100) | - |
| *sinH* | 27 (100) | 23 (100) | - |
| *uclD* | 24 (89) | 21 (91) | 1 |
| *agn43* | 13 (48) | 21 (91) | 1 |
| *papG* III | 11 (41) | 9 (39) | 0.908 |
| *focG* | 22 (82) | 17 (74) | 0.52 |
| *sfaG* | 8 (30) | 13 (57) | 0.055 |
| *papG* II *** | 17 (63) | 0 (0) | <0.001 |
| *papG* I | 0 (0) | 1 (4) | 0.46 |
| *afaE* | 0 (0) | 0 (0) | - |
| **Other:** | | | |
| *kpsD*, *kpsM* | 27 (100) | 23 (100) | - |
| *hlyF* | 0 (0) | 1 (4) | 0.46 |

* gene whose association with animal or human host was considered significant at *p*-value ≤ 0.05.
