# Supplementary material for: Zoonotic potential of uropathogenic Escherichia coli lineages from companion animals
Source: Vet Res. 2025 Mar 26;56:69. doi: 10.1186/s13567-025-01493-0 (PMC11948896; doi:10.1186/s13567-025-01493-0)
Supplement: Supplementary file 3 — Additional file 3. Distribution (%) of antimicrobial resistance phenotypes among urinary E. coli strains isolated from animals having received (ATB+) or not (ATB-) an antibiotic treatment during the past six months. Pen, penicillins; Ceph, cephalosporins; C1G/C2G, cephalosporins of first and second generations; C3G/C4G, cephalosporins of third and fourth generations; QL, quinolones; AMG, aminoglycosides, SXT, co-trimoxazole; Fos, fosfomycin; NFT, nitrofurantoin. [file 13567_2025_1493_MOESM3_ESM.docx]

**Additional file 3 Distribution (%) of antimicrobial resistance phenotypes among urinary *E. coli* strains isolated from animals having received (ATB+) or not (ATB-) an antibiotic treatment during the past six months.**

Pen, penicillins; Ceph, cephalosporins; C1G/C2G, cephalosporins of first and second generations; C3G/C4G, cephalosporins of third and fourth generations; QL, quinolones; AMG, aminoglycosides, SXT, co-trimoxazole; Fos, fosfomycin; NFT, nitrofurantoin.
