# Supplementary material for: Zoonotic potential of uropathogenic Escherichia coli lineages from companion animals
Source: Vet Res. 2025 Mar 26;56:69. doi: 10.1186/s13567-025-01493-0 (PMC11948896; doi:10.1186/s13567-025-01493-0)
Supplement: Supplementary file 4 — Additional file 4. Distribution (%) of major serotypes among urinary E. coli ST73 strains isolated from humans (A) and companion animals (B), respectively. [file 13567_2025_1493_MOESM4_ESM.docx]

**Additional file 4 Distribution (%) of major serotypes among urinary *E. coli* ST73 strains isolated from humans (A) and companion animals (B), respectively**.

**A Humans**

**B Companion animals**
