# Supplementary material for: Zoonotic potential of uropathogenic Escherichia coli lineages from companion animals
Source: Vet Res. 2025 Mar 26;56:69. doi: 10.1186/s13567-025-01493-0 (PMC11948896; doi:10.1186/s13567-025-01493-0)
Supplement: Supplementary file 5 — Additional file 5. Distribution (%) of virulence-associated genes among urinary E. coli strains isolated from 225 humans, 44 cats and 91 dogs. *, the difference between human and companion animal E. coli strains was considered significant at p-value <0.05. [file 13567_2025_1493_MOESM5_ESM.docx]

**Additional file 5 Distribution (%) of virulence-associated genes among urinary *E. coli* strains isolated from 225 humans, 44 cats and 91 dogs.**


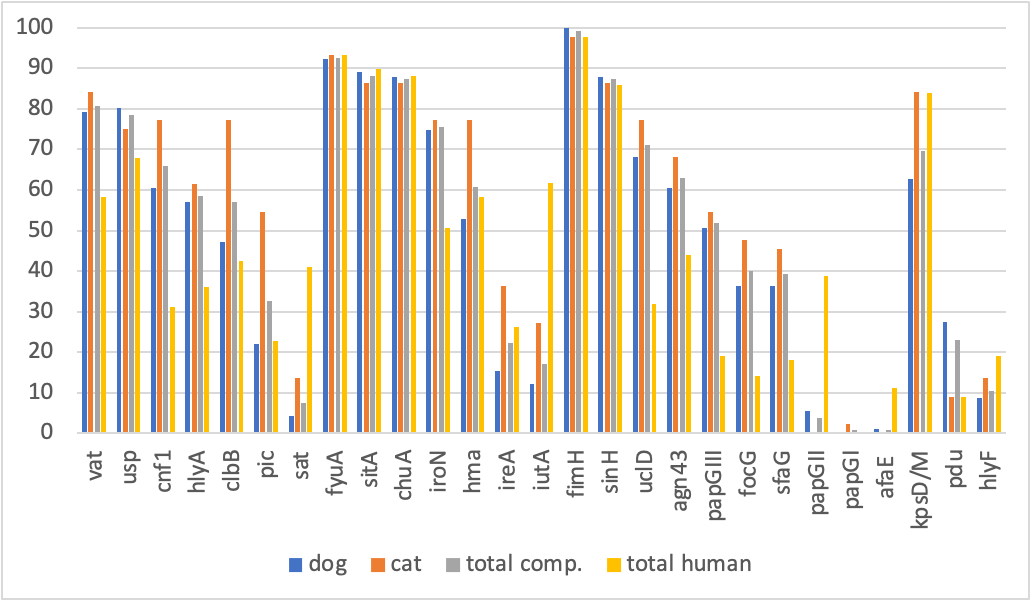


*

*

*

*

*

*

*

*

*

*

*

*

*

*

*

*

*

*

*

* the difference between human and companion animal *E. coli* strains was considered significant at *p*-value <0.05.
