# Supplementary material for: Zoonotic potential of uropathogenic Escherichia coli lineages from companion animals
Source: Vet Res. 2025 Mar 26;56:69. doi: 10.1186/s13567-025-01493-0 (PMC11948896; doi:10.1186/s13567-025-01493-0)
Supplement: Supplementary file 6 — Additional file 6. Distribution (%) of sequence types among 225 and 135 urinary E. coli isolates from humans (A) and companion animals (B), respectively. [file 13567_2025_1493_MOESM6_ESM.docx]

**Additional file 6 Distribution (%) of sequence types among 225 and 135 urinary *E. coli* isolates from humans (A) and companion animals (B), respectively.**

**A Humans**

**B Companion animals**
